# Brunn–Minkowski Inequality for $\theta$-Convolution Bodies via Ball’s Bodies

**DOI:** 10.1007/s12220-023-01508-2

**Published:** 2023-12-29

**Authors:** David Alonso-Gutiérrez, Javier Martín Goñi

**Affiliations:** 1 Área de análisis matemático, Departamento de matemáticas, Facultad de Ciencias, Universidad de Zaragoza, IUMA, Pedro Cerbuna 12, 50009 Zaragoza, Spain; 2 Faculty of Computer Science and Mathematics, University of Passau, Innstrasse 33, 94032 Passau, Germany

**Keywords:** Brunn–Minkowski inequality, $\theta$-Convolution bodies, Ball‘s bodies, 52A40, 52A420

## Abstract

We consider the problem of finding the best function $\varphi _n:[0,1]\rightarrow {\mathbb {R}}$ such that for any pair of convex bodies $K,L\in {\mathbb {R}}^n$ the following Brunn–Minkowski type inequality holds

$$\begin{aligned} |K+_\theta L|^\frac{1}{n}\ge \varphi _n(\theta )(|K|^\frac{1}{n}+|L|^\frac{1}{n}), \end{aligned}$$

where $K+_\theta L$ is the $\theta$-convolution body of *K* and *L*. We prove a sharp inclusion of the family of Ball’s bodies of an $\alpha$-concave function in its super-level sets in order to provide the best possible function in the range $\left( \frac{3}{4}\right) ^n\le \theta \le 1$, characterizing the equality cases.

## Introduction

It is well known that for any pair of convex bodies (i.e., compact convex sets with non-empty interior) $K,L\subseteq {\mathbb {R}}^n$, their Minkowski sum $K+L$, defined as

$$\begin{aligned} K+L:=\{x+y:x\in K, y\in L\}=\{z\in {\mathbb {R}}^n:\,K\cap (z-L)\ne \emptyset \}, \end{aligned}$$

is a convex body whose volume (or *n*-dimensional Lebesgue measure) $|\cdot |$ verifies, by Brunn–Minkowski inequality (see [8, Theorem 7.1.1]), that

1.1 $$\begin{aligned} |K+L|^\frac{1}{n}\ge |K|^\frac{1}{n}+|L|^\frac{1}{n}. \end{aligned}$$

In [2], the authors considered, for any $0\le \theta \le 1$ the $\theta$-convolution bodies of a pair of convex bodies $K,L\subseteq {\mathbb {R}}^n$, defined as

$$\begin{aligned} K+_\theta L:=\{z\in K+L:|K\cap (z-L)|\ge \theta M(K,L)\}, \end{aligned}$$

where $M(K,L)=\max _{z\in {\mathbb {R}}^n}|K\cap (z-L)|$, and studied the problem of obtaining the best possible function $\varphi _n:[0,1]\rightarrow {\mathbb {R}}$ such that for any pair of convex bodies $K,L\subseteq {\mathbb {R}}^n$ one has the following Brunn–Minkowski type inequality

1.2 $$\begin{aligned} |K+_\theta L|^\frac{1}{n}\ge \varphi _n(\theta )(|K|^\frac{1}{n}+|L|^\frac{1}{n}). \end{aligned}$$

The authors proved that for any pair of convex bodies $\frac{K+_\theta L}{1-\theta ^\frac{1}{n}}$ is an increasing family of convex bodies in $\theta$ and, as a consequence of Brunn–Minkowski inequality,

$$\begin{aligned} \varphi _n(\theta )\ge 1-\theta ^\frac{1}{n} \end{aligned}$$

for every $\theta \in [0,1]$. Therefore, for every pair of convex bodies $K,L\subseteq {\mathbb {R}}^n$

1.3 $$\begin{aligned} |K+_\theta L|^\frac{1}{n}\ge (1-\theta ^\frac{1}{n})(|K|^\frac{1}{n}+|L|^\frac{1}{n}). \end{aligned}$$

It was also shown in [2] that the increasing sequence of convex bodies $\frac{K+_\theta L}{1-\theta ^\frac{1}{n}}$ remains constant if and only if $K=-L$ is an *n*-dimensional simplex, in which case there is no equality in Brunn–Minkowski inequality (1.1). Therefore, inequality (1.3) is not sharp. We are going to improve the estimate of the function $\varphi _n$, proving a sharp analogue of (1.3) in the range $\left( \frac{3}{4}\right) ^n\le \theta \le 1$. Namely, we will prove the following:

### Theorem 1.1

Let $K,L\subseteq {\mathbb {R}}^n$ be convex bodies. Then, for every $\left( \frac{3}{4}\right) ^n\le \theta \le 1$ we have that

$$\begin{aligned} |K+_\theta L|^\frac{1}{n}\ge \frac{1}{2}{{2n}\atopwithdelims (){n}}^\frac{1}{n}(1-\theta ^\frac{1}{n})\left( |K|^\frac{1}{n}+|L|^\frac{1}{n}\right) \end{aligned}$$

and, for every $0\le \theta \le \left( \frac{3}{4}\right) ^n$,

$$\begin{aligned} |K+_\theta L|^\frac{1}{n}\ge \left( 1-\left( \frac{4}{3}-\frac{1}{6}{{2n}\atopwithdelims (){n}}^\frac{1}{n}\right) \theta ^\frac{1}{n}\right) \left( |K|^\frac{1}{n}+|L|^\frac{1}{n}\right) . \end{aligned}$$

Moreover, given any $\left( \frac{3}{4}\right) ^n\le \theta <1$ there is equality if and only if $K=-L$ is an *n*-dimensional simplex.

### Remark

The constant $\frac{1}{2}{{2n}\atopwithdelims (){n}}^\frac{1}{n}$ is asymptotically 2, so the result improves on (1.3) for values of $\theta$ close to 1, in which case there is equality whenever $K=-L$ is an *n*-dimensional simplex. Besides, $\left( \frac{4}{3}-\frac{1}{6}{{2n}\atopwithdelims (){n}}^\frac{1}{n}\right)$ is asymptotically $\frac{2}{3}$. Therefore, this result also improves on (1.3), for small values of $\theta$.

### Remark

The change of behavior in the estimate at $\theta _0=\left( \frac{3}{4}\right) ^n$ is due to the method we use in order to prove Theorem 1.1. However, it is clear that the estimate obtained for the function $\varphi _n(\theta )$ for $\theta _0\le \theta \le 1$ cannot hold in the whole range $0\le \theta \le 1$, as this would lead, taking limit as $\theta$ tends to 0, to Brunn–Minkowski’s inequality (1.1) with an extra factor $\frac{1}{2}{{2n}\atopwithdelims (){n}}^\frac{1}{n}$ which is asymptotically 2 and such inequality is false if $K=L$. We do not have a conjecture on what the behavior of $\varphi _n(\theta )$ is for $0\le \theta \le \theta _0$.

In order to prove Theorem 1.1 we observe that the $\theta$-convolution bodies of two convex bodies $K,L\subseteq {\mathbb {R}}^n$ are the super-level sets of the function ${\tilde{g}}_{K,L}: {\mathbb {R}}^n\rightarrow [0,1]$ given by ${\tilde{g}}_{K,L}(z)=\frac{|K\cap (z-L)|}{M(K,L)}$, which is $\frac{1}{n}$-concave (i.e., ${\tilde{g}}_{K,L}^\frac{1}{n}$ is concave on its support, $K+L$) and, in particular, is log-concave. Given any integrable log-concave function $g:{\mathbb {R}}^n\rightarrow [0,\infty )$ (i.e., $\log g:{\mathbb {R}}^n\rightarrow [-\infty ,\infty )$ is concave) with $g(0)\ne 0$, Ball [3] defined the following family of convex bodies associated to it $(K_p(g))_{p>0}$:

$$\begin{aligned} K_p(g):=\left\{ x\in {\mathbb {R}}^n:\,p\int _0^\infty r^{p-1}g(rx)dr\ge g(0)\right\} . \end{aligned}$$

In [1, Lemma 2.3.2, Remark 2.6], the authors proved, following the ideas in [6], the following inclusion relation between Ball’s bodies and super-level sets of a log-concave function in a certain range of the parameters involved: If $g:{\mathbb {R}}^n\rightarrow [0,\infty )$ is an integrable log-concave function with $\Vert g\Vert _\infty =g(0)$, then for any $p>0$ and any $0<t<\frac{p}{e}$

1.4 $$\begin{aligned} \frac{t}{\Gamma (1+p)^\frac{1}{p}}K_p(g)\subseteq \{x\in {\mathbb {R}}^n:\,g(x)\ge e^{-t}\Vert g\Vert _\infty \}. \end{aligned}$$

We will obtain a sharper inclusion relation between the family Ball’s bodies and the super-level sets of an $\alpha$-concave function *g* (i.e., $g^\alpha$ is concave on its support) which will allow us to prove Theorem 1.1. We will denote, for any $0\le r\le 1$, by $L_r$ the super-level set of an $\alpha$-concave function *g* given by

1.5 $$\begin{aligned} L_r(g)= 
& {} \{x\in \text {supp}(g):\,g^\alpha (x)\ge r\Vert g\Vert _\infty ^\alpha \} \nonumber \\= & {} \{x\in \text {supp}(g):\,g(x)\ge r^{\frac{1}{\alpha }}\Vert g\Vert _\infty \}. \end{aligned}$$

The boundary of the convex body $K\subseteq {\mathbb {R}}^n$ containing the origin in its interior and its radial function (see definition below) will be denoted by $\partial K$ and by $\rho _K$. Besides, for any $x,y>0$ the generalized binomial coefficient ${x\atopwithdelims ()y}$ is defined in terms of the gamma function as $\displaystyle { {x\atopwithdelims ()y}:= \frac{\Gamma {(1+x)}}{\Gamma {(1+y)}\Gamma {(1+x-y)}}}$. Using this notation, we will prove the following:

### Theorem 1.2

Let $p>0$ and let $K\subseteq {\mathbb {R}}^n$ be a convex body with $0\in K$ and let $g:K\rightarrow [0,\infty )$ be a continuous $\alpha$-concave function, with $\alpha >0$, such that $\Vert g\Vert _\infty =g(0)>0$ and such that $g(x)=0$ for every $x\in \partial K$. Then, understanding that $g(x)=0$ for every $x\not \in K$, we have that for every $t\in \left[ 0,\frac{p\alpha }{(1+p\alpha )^{1+\frac{1}{p\alpha }}}\right]$

$$\begin{aligned} t{{p+\frac{1}{\alpha }}\atopwithdelims (){p}}^\frac{1}{p}K_p(g)\subseteq L_{1-t}(g). \end{aligned}$$

Moreover, for any $t\in \left( 0,\frac{p\alpha }{(1+p\alpha )^{1+\frac{1}{p\alpha }}}\right]$ there is equality if and only if $g(x)=\Vert g\Vert _\infty \left( 1-\Vert x\Vert _K\right) ^\frac{1}{\alpha }$ for every $x\in K$. Furthermore, given $u\in S^{n-1}$, if there exists $r\in [0,\rho _K(u)]$ such that $g(ru)\ne \Vert g\Vert _\infty \left( 1-\Vert ru\Vert _K\right) ^\frac{1}{\alpha }$, then, there exists $\varepsilon >0$ such that for every $t\in \left( 0,\frac{p\alpha }{(1+p\alpha )^{1+\frac{1}{p\alpha }}}\right]$

$$\begin{aligned} t{{p+\frac{1}{\alpha }}\atopwithdelims (){p}}^\frac{1}{p}(\rho _{K_p(g)}(u)+\varepsilon )\le \rho _{L_{1-t}(g)}(u). \end{aligned}$$

The paper is organised as follows. In Sect. 2 we will provide the necessary definitions and results that we need to prove our results. In Sect. 3 we will provide the proof of Theorem 1.2. Finally, in Sect. 4 we will prove Theorem 1.1.

## Preliminaries

### Notation

Given a convex body $K\subseteq {\mathbb {R}}^n$ with $0\in \text {int}K$, we will denote by $\rho _K$ the radial function $\rho _K:S^{n-1}\rightarrow [0,\infty )$ given by $\rho _K(u)=\max \{\lambda \ge 0:\,\lambda u\in K\}$, where $S^{n-1}$ denotes the $(n-1)$-dimensional Euclidean sphere in ${\mathbb {R}}^n$, that is, $S^{n-1}=\{u\in {\mathbb {R}}^n:\,\Vert u\Vert _2=1\}$. It is well known that given two convex bodies $K_1,K_2\in {\mathbb {R}}^n$ we have that $K_1\subseteq K_2\Leftrightarrow \rho _{K_1}(u)\le \rho _{K_2}(u)$ for every $u\in S^{n-1}$. The Minkowski gauge of *K* is defined as

$$\begin{aligned} \Vert x\Vert _K=\inf \{\lambda >0:\,x\in \lambda K\}. \end{aligned}$$

Notice that for every $u\in S^{n-1}$ we have that $\Vert u\Vert _K=\frac{1}{\rho _K(u)}$. $\chi _K$ will denote the characteristic function of a convex body *K* and $K-K$ will always denote the difference body $K+(-K)$. Whenever $K\subseteq {\mathbb {R}}^n$ is a *k*-dimensional set contained in an affine *k*-dimensional subspace, |*K*| will denote its *k*-dimensional Lebesgue measure. $B_2^n$ will stand for the Euclidean unit ball, $\Vert \cdot \Vert _2$ for the Euclidean norm and $\Delta ^n$ will stand for the regular simplex.

### Ball’s Bodies

A log-concave function $g:{\mathbb {R}}^n\rightarrow [0,\infty )$ is a function of the form $g(x)=e^{-u(x)}$ with $u:{\mathbb {R}}^n\rightarrow (-\infty ,\infty ]$ a convex function. The family of log-concave functions plays an extremely important role in the study of problems related to distribution of volume in convex bodies. In [3], Ball introduced a family of convex bodies $(K_p(g))_{p>0}$ associated to log-concave functions verifying $g(0)>0$. More precisely, for any measurable (not necessarily log-concave) $g:{\mathbb {R}}^n\rightarrow [0,\infty )$ such that $g(0)>0$ and $p>0$, $K_p(g)$ is defined as

$$\begin{aligned} K_p(g):=\left\{ x\in {\mathbb {R}}^n:\,p\int _0^\infty r^{p-1}g(rx)dr\ge g(0)\right\} . \end{aligned}$$

$K_p(g)$ is a star set with center 0 whose radial function is given by

$$\begin{aligned} \rho _{K_p(g)}^p(u)=\frac{p}{g(0)}\int _0^\infty r^{p-1}g(ru)dr,\quad \forall u\in S^{n-1}. \end{aligned}$$

It is well known that for any integrable log-concave function with $g(0)>0$, $K_p(g)$ is convex for every $p>0$ and, by integration in polar coordinates, we have that $\displaystyle {|K_n(g)|=\int _{{\mathbb {R}}^n}\frac{g(x)}{g(0)}dx}$. We refer the reader to [4, Section 2.5] for more information on Ball’s bodies.

### The Generalized Covariogram Function

Given a convex body $K\subseteq {\mathbb {R}}^n$, its covariogram function is the function $g_K:K-K\rightarrow [0,\infty )$ given by

$$\begin{aligned} g_K(z)=|K\cap (z+K)|. \end{aligned}$$

Through this paper we will consider, given a pair of convex bodies $K,L\subseteq {\mathbb {R}}^n$, the generalized covariogram function defined as $g_{K,L}:K+L\rightarrow [0,\infty )$ given by

$$\begin{aligned} g_{K,L}(z)=|K\cap (z-L)|=\chi _K*\chi _L(z), \end{aligned}$$

where $\chi _K*\chi _L$ denotes the convolution of the functions $\chi _K$ and $\chi _L$. Notice that for any convex body $g_K=g_{K,-K}$. As a consequence of Brunn–Minkowski inequality (1.1), we have that for any pair of convex bodies $K,L\subseteq {\mathbb {R}}^n$, $g_{K,L}$ is $\frac{1}{n}$-concave and then, for any $\theta \in [0,1]$ we have that

$$\begin{aligned} K+_\theta L=L_{\theta ^\frac{1}{n}}(g_{K,L}), \end{aligned}$$

where $L_{\theta ^\frac{1}{n}}(g_{K,L})$ is defined by (1.5). Besides, (see [2, Proposition 2.1]) for any $x\in {\mathbb {R}}^n$ and any $\theta \in [0,1]$

2.1 $$\begin{aligned} (x+K)+_\theta L=x+(K+_\theta L) \end{aligned}$$

and for any pair of convex bodies $K,L\subseteq {\mathbb {R}}^n$ one has that

$$\begin{aligned} \int _{{\mathbb {R}}^n}\frac{g_{K,L}(z)}{\Vert g_{K,L}\Vert _\infty }dz=\frac{|K||L|}{M(K,L)}. \end{aligned}$$

It was also proved in [2] that $\frac{K+_\theta L}{1-\theta ^\frac{1}{n}}$ is an increasing family of convex bodies in $\theta \in [0,1]$ and (see [2, Proposition 2.8]) that $K+_\theta L=(1-\theta ^\frac{1}{n})(K+L)$ for every $0\le \theta \le 1$ if and only if $K=-L$ is a simplex.

### The Polar Projection Body and Zhang’s Inequality

Given a convex body $K\subseteq {\mathbb {R}}^n$, its polar projection body $\Pi ^*K$ is defined as the unit ball of the norm given by

$$\begin{aligned} \Vert x\Vert _{\Pi ^*K}=\Vert x\Vert _2|P_{x^\perp }K|. \end{aligned}$$

It is well known that for any convex body, $|K|^{n-1}|\Pi ^*K|$ is an affine invariant quantity that verifies Petty projection inequality [7] (also known as the affine isoperimetric inequality):

$$\begin{aligned} |K|^{n-1}|\Pi ^*K|\le |B_2^n|^{n-1}|\Pi ^*B_2^n|, \end{aligned}$$

with equality if and only if *K* is an ellipsoid. In [10], Zhang proved the following reverse inequality for any convex body $K\subseteq {\mathbb {R}}^n$:

2.2 $$\begin{aligned} |K|^{n-1}|\Pi ^*K|\ge |\Delta ^n|^{n-1}|\Pi ^*\Delta ^n|=\frac{1}{n^n}{{2n}\atopwithdelims (){n}}, \end{aligned}$$

with equality if and only if *K* is a simplex (see also [5] for another proof).

In [9], Tsolomitis studied the existence and the behavior of limiting convolution bodies

$$\begin{aligned} C_\alpha (K,L):=\lim _{\theta \rightarrow 1^-}\frac{K+_\theta L}{(1-\theta )^\alpha } \end{aligned}$$

for symmetric convex bodies *K* and *L*, and some exponent $\alpha$. He also gave some regularity conditions under which the above limit is non-degenerated for some specific value $\alpha$. Taking into account that for any convex body $K\subseteq {\mathbb {R}}^n$, $C_1(K,-K)=|K|\Pi ^*K$, in [2, Theorem 4.6], the authors showed that Zhang’s inequality can be extended to

2.3 $$\begin{aligned} |C_1(K,L)|\ge \frac{1}{n^n}{{2n}\atopwithdelims (){n}}\frac{|K||L|}{M(K,L)}, \end{aligned}$$

for any pair of convex bodies $K,L\subseteq {\mathbb {R}}^n$ such that $M(K,L)=|K\cap (-L)|$, with equality if and only if $K=-L$ is a simplex.

## An Inclusion Relation Between Ball’s Bodies and Superlevel Sets

In this section we are going to prove Theorem 1.2, from which we will derive Theorem 1.1 in the following section.

### Proof of Theorem 1.2

Let us assume, without loss of generality that $\Vert g\Vert _\infty =g(0)=1$. Otherwise, consider the function $\frac{g}{\Vert g\Vert _\infty }$. Let us denote, for any $u\in S^{n-1}$, $l_u=\sup \{r>0\,:\,g(ru)>0\}=\rho _K(u)$, $v_u:[0,l_u]\rightarrow [0,1]$ the function defined as $v_u(r)=g^\alpha (ru)$, which is concave on $[0,l_u]$, and, for any $q>0$, let $\phi _u:[0,l_u]\rightarrow [0,\infty )$ the function defined as

$$\begin{aligned} \phi _u(r)=r^{q\alpha }v_u(r)=r^{q\alpha }g^\alpha (ru). \end{aligned}$$

Notice that since $\log \phi _u$ is strictly concave on $(0,l_u)$, $\displaystyle {\lim _{r\rightarrow 0^+}\log \phi _u(r)=-\infty }$, and $\displaystyle {\lim _{r\rightarrow l_u^-}\log \phi _u(r)=-\infty }$, $\phi _u$ attains a unique maximum at some $r_0=r_0(q)\in (0,l_u)$. Therefore, denoting by $(\phi _u)_{-}(r_0)$ and $(\phi _u)_{+}(r_0)$ the lateral derivatives of $\phi _u$ at $r_0$ we have that

- $$0\le (\phi _u)_{-}(r_0)=r_0^{q\alpha }\left( \frac{q\alpha }{r_0}v_u(r_0)+(v_u)_{-}(r_0)\right) $$,
- $$0\ge (\phi _u)_{+}(r_0)=r_0^{q\alpha }\left( \frac{q\alpha }{r_0}v_u(r_0)+(v_u)_{+}(r_0)\right) $$.

Then,

- $$(v_u)_{-}(r_0)\ge -\frac{q\alpha }{r_0}v_u(r_0)$$,
- $$(v_u)_{+}(r_0)\le -\frac{q\alpha }{r_0}v_u(r_0)$$.

Notice that if $v_u$ is an affine function then necessarily for every $q>0$ we have that $v_u(r)=v_u(r_0)\left( 1-\frac{q\alpha }{r_0}(r-r_0)\right)$. We will denote this affine function by

$$\begin{aligned} \tau _{u,q}(r)=v_u(r_0(q))\left( 1-\frac{q\alpha }{r_0(q)}(r-r_0(q))\right) ,\quad \forall r\in [0,l_u]. \end{aligned}$$

For any $q>0$, the graph of the function $\tau _{u,q}$ is a supporting line of the hypograph of $v_u$ and then

$$\begin{aligned} v_u(r)\le \tau _{u,q}(r)=v_u(r_0)\left( 1-\frac{q\alpha }{r_0}(r-r_0)\right) ,\quad \forall r\in [0,l_u]. \end{aligned}$$

Thus, for any $p,q>0$

3.1 $$\begin{aligned} \rho _{K_p(g)}^p(u)= & {} p\int _0^{\infty }r^{p-1}g(ru)dr=p\int _0^{l_u}r^{p-1}v_u^\frac{1}{\alpha }(r)dr\nonumber \\\le & {} pv_u^\frac{1}{\alpha }(r_0)\int _0^{l_u}r^{p-1}\left( 1-\frac{q\alpha }{r_0}(r-r_0)\right) ^\frac{1}{\alpha }dr\nonumber \\\le & {} pg(r_0u)\int _0^{\left( 1+\frac{1}{q\alpha }\right) r_0}r^{p-1}\left( 1-\frac{q\alpha }{r_0}(r-r_0)\right) ^\frac{1}{\alpha }dr\nonumber \\= & {} pg(r_0u)\left( 1+q\alpha \right) ^\frac{1}{\alpha }\left( 1+\frac{1}{q\alpha }\right) ^pr_0^p\int _0^1s^{p-1}\left( 1-s\right) ^\frac{1}{\alpha }ds\nonumber \\= & {} pg(r_0u)\frac{\left( 1+q\alpha \right) ^{p+\frac{1}{\alpha }}}{(q\alpha )^p}r_0^p\beta \left( p,1+\frac{1}{\alpha }\right) . \end{aligned}$$

Moreover, for any $q>0$, the previous inequality is an equality if and only if $l_u=\left( 1+\frac{1}{q\alpha }\right) r_0$ and $v_u(r)=\tau _{u,q}(r)$ for every $0\le r\le l_u=\left( 1+\frac{1}{q\alpha }\right) r_0$. That is, if $v_u$ is an affine function such that $v_u(l_u)=0$.

Consequently, for any $p,q>0$

$$\begin{aligned} \frac{q\alpha }{\left( 1+q\alpha \right) ^{1+\frac{1}{p\alpha }}}{{p+\frac{1}{\alpha }}\atopwithdelims (){p}}^\frac{1}{p}\rho _{K_p(g)}(u)\le g(r_0u)^\frac{1}{p}r_0, \end{aligned}$$

with equality if and only if $v_u$ is an affine function such that $v_u(l_u)=0$.

On the one hand, since $v_u$ is concave on $[0, l_u]$ and $\Vert g\Vert _\infty =g(0)=1$, we have that

$$\begin{aligned} g^\alpha \left( g^\frac{1}{p}(r_0u)r_0u\right)= & {} v_u\left( g^\frac{1}{p}(r_0u)r_0\right) \ge g^\frac{1}{p}(r_0u)v_u(r_0)+\left( 1- g^\frac{1}{p}(r_0u)\right) v_u(0)\\= & {} g^\frac{1}{p}(r_0u)g^\alpha (r_0u)+1- g^\frac{1}{p}(r_0u)\\= & {} 1-\left( g^\alpha (r_0u)\right) ^\frac{1}{p\alpha }\left( 1-g^\alpha (r_0u)\right) , \end{aligned}$$

with equality if and only if $v_u$ is affine on $[0,r_0]$ and then $v_u(r)=\tau _{u,q}(r)$ for every $0\le r\le r_0$. On the other hand, since $v_u$ is concave on $[0, l_u]$, we have that $(v_u)_-$ is decreasing on $[0, l_u]$ and then

$$\begin{aligned} v_u(r_0)= & {} v_u(0)+\int _0^{r_0}(v_u)_-(r)dr\ge v_u(0)+(v_u)_-(r_0)r_0\ge v_u(0)-q\alpha v_u(r_0)\\= & {} 1-q\alpha v_u(r_0). \end{aligned}$$

Therefore,

$$\begin{aligned} g^\alpha (r_0u)=v_u(r_0)\ge \frac{1}{1+q\alpha }, \end{aligned}$$

with equality if and only if $(v_u)_-=-\frac{q\alpha }{r_0} v_u(r_0)$ for every $0\le r<r_0$, in which case $v_u(r)=\tau _{u,q}(r)$ for every $0\le r< r_0$. Since the function $h(x)=1-x^\frac{1}{p\alpha }(1-x)$ is decreasing in $\left[ 0,\frac{1}{1+p\alpha }\right]$ and is increasing in $\left[ \frac{1}{1+p\alpha },1\right]$, we have that if $\frac{1}{1+p\alpha }\le \frac{1}{1+q\alpha }$ (which happens whenever $0<q\le p$)

$$\begin{aligned} g^\alpha \left( g^\frac{1}{p}(r_0u)r_0u\right)\ge & {} 1-\left( g^\alpha (r_0u)\right) ^\frac{1}{p\alpha }\left( 1-g^\alpha (r_0u)\right) =h(g^\alpha (r_0u))\ge h\left( \frac{1}{1+q\alpha }\right) \\= & {} 1-\left( \frac{1}{1+q\alpha }\right) ^\frac{1}{p\alpha }\left( 1-\frac{1}{1+q\alpha }\right) \\= & {} 1-\frac{q\alpha }{(1+q\alpha )^{1+\frac{1}{p\alpha }}}. \end{aligned}$$

Besides, there is equality if and only if $v_u(r)=\tau _{u,q}(r)$ for every $0\le r\le r_0$ and

$$\begin{aligned} g^\alpha (r_0u)=v_u(r_0)=\frac{1}{1+q\alpha }, \end{aligned}$$

which also happens if and only if $v_u(r)=\tau _{u,q}(r)$ for every $0\le r< r_0$.

Therefore, calling $t(p,q,\alpha ):=\frac{q\alpha }{(1+q\alpha )^{1+\frac{1}{p\alpha }}}$, if $0<q\le p$, we have that $g^\frac{1}{p}(r_0u)r_0u\in L_{1-t(p,q,\alpha )}(g)$ and, since $0\in L_{1-t(p,q,\alpha )}(g)$ as $\Vert g\Vert _\infty =g(0)=1$ and $L_{1-t(p,q,\alpha )}(g)$ is convex, we have that

$$\begin{aligned} g^\frac{1}{p}(r_0u)r_0\le \rho _{L_{1-t(p,q,\alpha )}}(u), \end{aligned}$$

with equality if and only if $g^\frac{1}{p}(r_0u)r_0u\in \partial L_{1-t(p,q,\alpha )}$, which happens if and only if $v_u(r)=\tau _{u,q}(r)$ for every $0\le r\le r_0$. Thus, if $0<q\le p$

$$\begin{aligned} t(p,q,\alpha ){{p+\frac{1}{\alpha }}\atopwithdelims (){p}}^\frac{1}{p}\rho _{K_p(g)}(u)\le \rho _{L_{1-t(p,q,\alpha )}}(u), \end{aligned}$$

with equality if and only $v_u(r)=\tau _{u,q}(r)$ for every $0\le r\le l_u=\left( 1+\frac{1}{q\alpha }\right) r_0$, i.e., if $v_u$ is an affine function such that $v_u(l_u)=0$. Since this happens for every $u\in S^{n-1}$,

$$\begin{aligned} t(p,q,\alpha ){{p+\frac{1}{\alpha }}\atopwithdelims (){p}}^\frac{1}{p}K_p(g)\subseteq L_{1-t(p,q,\alpha )}(g) \end{aligned}$$

and, for any $$0<q\le p$$, there is equality equality if and only if for every direction $$u\in 
S^{n-1}$$ $$v_u$$ is an affine function such that $$v_u(l_u)=0$$. That is, if $$g(x)=\Vert g\Vert _\infty \left( 1-\Vert x\Vert _K\right) ^\frac{1}{\alpha }$$ for every $$x\in K$$. Since the function $$h_1(x)=\frac{x}{(1+x)^{1+\frac{1}{p\alpha }}}$$ is continuous and increasing in $$[0,p\alpha ]$$, it attains every value in $$\left[ 0,t(p,p,\alpha )\right] $$, where $$t(p,q,\alpha )$$ lies if $$0<q\le p$$. Therefore, we have that for every $$t\in \left[ 0,t(p,p,\alpha )\right] $$.

$$\begin{aligned} t{{p+\frac{1}{\alpha }}\atopwithdelims (){p}}^\frac{1}{p}K_p(g)\subseteq L_{1-t}(g) \end{aligned}$$

and, for any $t\in \left[ 0,t(p,p,\alpha )\right]$, there is equality if and only if $g(x)=\Vert g\Vert _\infty \left( 1-\Vert x\Vert _K\right) ^\frac{1}{\alpha }$.

Assume now that for some $u\in S^{n-1}$ there exists $r\in [0,\rho _K(u)]$ such that $g(ru)\ne \Vert g\Vert _\infty \left( 1-\Vert ru\Vert _K\right) ^\frac{1}{\alpha }$. Therefore, $v_u$ is not an affine function.

We first notice that the function $r_0(q)$ is continuous on $q\in (0,\infty )$. Indeed, let $(q_k)_{k=1}^\infty \subseteq (0,\infty )$ be a sequence converging to some $q\in (0,\infty )$. Let $(q_{k_i})_{i=1}^\infty$ be any convergent subsequence of $(q_k)_{k=1}^\infty$ such that $r_0(q_{k_i})$ converges to some ${\overline{r}}\in [0,l_u]$, which exist since $(r_0(q_k))_{k=1}^\infty \subseteq (0,l_u)$. Since for every $i\in {\mathbb {N}}$, we have, by the definition of $r_0(q_{k_i})$, that

$$\begin{aligned} r_0(q)^{q_{k_i}}v_u(r_0(q))\le r_0(q_{k_i})^{q_{k_i}}v_u(r_0(q_{k_i})), \end{aligned}$$

taking limits as *i* tends to $\infty$ we obtain that

$$\begin{aligned} r_0(q)^{q}v_u(r_0(q))\le {\overline{r}}^{q}v_u({\overline{r}}). \end{aligned}$$

Therefore, by the definition of $r_0(q)$, ${\overline{r}}=r_0(q)$. Therefore,

$$\begin{aligned} \liminf _{k\rightarrow \infty }r_0(q_k)=\limsup _{k\rightarrow \infty }r_0(q_k)=r_0(q) \end{aligned}$$

and then $r_0(q_k)$ converges to $r_0(q)$. Thus $r_0(q)$ is continuous on $q\in (0,\infty )$. Besides, if $(q_k)_{k=1}^\infty \subseteq (0,\infty )$ is a sequence converging to 0 and for some subsequence $r_0(q_{k_i})$ converges to $l_u$ we would have that for every $r\in [0,l_u]$

$$\begin{aligned} v_u(r)\le \tau _{u,q_{k_i}}(r)=v_u(r_0(q_{k_i}))\left( 1-\frac{q_{k_i}\alpha }{r_0(q_{k_i})}(r-r_0(q_{k_i}))\right) , \end{aligned}$$

leading to $v_u(r)\le 0$, which is a contradiction. Therefore, for any $p>0$ we have that $s:=\sup \{r_0(q):\,q\in (0,p]\}<l_u$ and then, for every $p>0$ and every $0<q\le p$ we have that $\frac{q\alpha }{r_0(q)}v_u(r_0(q))\le -(v_u)_+(r_0(q))\le -(v_u)_+(s)$ and then $\frac{q\alpha }{r_0(q)}v_u(r_0(q))$ is bounded in $q\in (0,p]$.

Assume that there is no $\varepsilon >0$ such that for every $0<q\le p$ we have that

$$\begin{aligned} \varepsilon <p\int _0^{\left( 1+\frac{1}{q\alpha }\right) r_0(q)}r^{p-1}\tau _{u,q}^\frac{1}{\alpha }(r)dr-p\int _0^{l_u}r^{p-1}v_u^\frac{1}{\alpha }(r)dr. \end{aligned}$$

Then, we can find a sequence $(q_k)_{k=1}^\infty \subseteq (0,p]$ and, if necessary, extract from it further subsequences which we denote in the same way, such that

$$\begin{aligned} \lim _{k\rightarrow \infty }p\int _0^{\infty }r^{p-1}\left( \tau _{u,q_k}^\frac{1}{\alpha }(r)\chi _{\left[ 0,\left( 1+\frac{1}{q_k\alpha }\right) r_0(q_k)\right] }(r)-v_u^\frac{1}{\alpha }(r)\chi _{[0,l_u]}(r)\right) dr=0, \end{aligned}$$

$(q_k)_{k=1}^\infty$ converges to some $q\in [0,p]$, $r_0(q_k)$ converges to some ${\overline{r}}\in [0,l_u]$, and $\frac{q_k\alpha }{r_0(q_k)}v_u(r_0)$ converges to some $\lambda \in [0,\infty )$. Since for every $r\in [0,\infty )$ we have that for every $k\in {\mathbb {N}}$

$$\begin{aligned} v_u(r)\chi _{[0,l_u]}(r)\le & {} \tau _{u,q_k}(r)\chi _{\left[ 0,\left( 1+\frac{1}{q_k\alpha }\right) r_0(q_k)\right] }(r)\\= & {} v_u(r_0(q_k))\left( 1-\frac{q_k\alpha }{r_0(q_k)}(r-r_0(q_k))\right) \chi _{\left[ 0,\left( 1+\frac{1}{q_k\alpha }\right) r_0(q_k)\right] }(r), \end{aligned}$$

taking limits as $k\rightarrow \infty$ we obtain that for almost every $r\in [0,\infty )$

$$\begin{aligned} v_u(r)\chi _{[0,l_u]}(r)\le \left( v_u({\overline{r}})-\lambda (r-{\overline{r}})\right) \chi _{\left[ 0,\left( 1+\frac{1}{q\alpha }\right) {\overline{r}}\right] }(r) \end{aligned}$$

and then, by Fatou’s lemma,

$$\begin{aligned}&p\int _0^\infty r^{p-1}\left( \left( v_u({\overline{r}})-\lambda (r-{\overline{r}})\right) \chi _{\left[ 0,\left( 1+\frac{1}{q\alpha }\right) ^\frac{1}{\alpha }{\overline{r}}\right] }(r)-v_u(r)^\frac{1}{\alpha }\chi _{[0,l_u]}(r)\right) dr\\&\quad \le \lim _{k\rightarrow \infty }p\int _0^{\infty }r^{p-1}\left( \tau _{u,q_k}^\frac{1}{\alpha }(r)\chi _{\left[ 0,\left( 1+\frac{1}{q_k\alpha }\right) r_0(q_k)\right] }(r)-v_u^\frac{1}{\alpha }(r)\chi _{[0,l_u]}(r)\right) dr=0. \end{aligned}$$

Since the integrand in the first integral is non-negative, by continuity of the following functions, we have that for every $r\in [0,\infty )$

$$\begin{aligned} v_u(r)\chi _{[0,l_u]}(r)= \left( v_u({\overline{r}})-\lambda (r-{\overline{r}})\right) \chi _{\left[ 0,\left( 1+\frac{1}{q\alpha }\right) {\overline{r}}\right] }(r) \end{aligned}$$

and then $v_u$ is an affine function. Therefore, if $v_u$ is not linear, there exists ${\overline{\varepsilon }}>0$ such that for every $0<q\le p$

$$\begin{aligned} p\int _0^{l_u}r^{p-1}v_u^\frac{1}{\alpha }(r)dr+{\overline{\varepsilon }}\le pg(r_0u)\frac{\left( 1+q\alpha \right) ^{p+\frac{1}{\alpha }}}{(q\alpha )^p}r_0^p\beta \left( p,1+\frac{1}{\alpha }\right) \end{aligned}$$

and then there exists $\varepsilon >0$ such that for every $0<q\le p$

$$\begin{aligned} t(p,q,\alpha ){{p+\frac{1}{\alpha }}\atopwithdelims (){p}}^\frac{1}{p}\left( \rho _{K_p(g)}(u)+\varepsilon \right)\le & {} t(p,q,\alpha ){{p+\frac{1}{\alpha }}\atopwithdelims (){p}}^\frac{1}{p}\left( \rho _{K_p(g)}^p(u)+{\overline{\varepsilon }}\right) ^\frac{1}{p}\\\le & {} g(r_0u)^\frac{1}{p}r_0, \end{aligned}$$

Proceeding now as in the proof of the inequality we obtain the result. $\square$

## Brunn–Minkowski Inequality for $\theta$-Convolution Bodies

In this section we are going to prove Theorem 1.1.

### Proof of Theorem 1.1

Let $K,L\subseteq {\mathbb {R}}^n$ be a pair of convex bodies. By (2.1) we can assume, without loss of generality, that

$$\begin{aligned} M(K,L)=\max _{z\in {\mathbb {R}}^n}|K\cap (z-L)|=|K\cap (-L)|. \end{aligned}$$

Then, the function $g_{K,L}:K+L\rightarrow [0,\infty )$ given by $g_{K,L}(z)=|K\cap (z-L)|$, which is a continuous $\frac{1}{n}$-concave function, verifies that $\Vert g_{K,L}\Vert _\infty =g_{K,L}(0)$ and $g_{K,L}(z)=0$ for every $z\in \partial (K+L)$.

By Theorem 1.2 with $p=n$, we have that for every $0\le t\le \frac{1}{4}$

$$\begin{aligned} t{{2n}\atopwithdelims (){n}}^\frac{1}{n}K_n(g_{K,L})\subseteq L_{1-t}(g_{K,L}). \end{aligned}$$

Equivalently, taking $t=1-\theta ^\frac{1}{n}$ we have that if $\left( \frac{3}{4}\right) ^n\le \theta <1$

$$\begin{aligned} {{2n}\atopwithdelims (){n}}^\frac{1}{n}K_n(g_{K,L})\subseteq \frac{L_{\theta ^\frac{1}{n}}(g_{K,L})}{1-\theta ^\frac{1}{n}}=\frac{K+_\theta L}{1-\theta ^\frac{1}{n}}. \end{aligned}$$

Taking volumes, and noticing that

$$\begin{aligned} |K_n(g_{K,L})|=\int _{{\mathbb {R}}^n}\frac{g_{K,L}(x)}{g_{K,L}(0)}dx=\int _{{\mathbb {R}}^n}\frac{ g_{K,L}(x)}{\Vert g_{K,L}\Vert _\infty }dx=\frac{|K||L|}{M(K,L)} \end{aligned}$$

we obtain that for every $\left( \frac{3}{4}\right) ^n\le \theta <1$

$$\begin{aligned} \left| \frac{K+_\theta L}{1-\theta ^\frac{1}{n}}\right| ^\frac{1}{n}\ge & {} {{2n}\atopwithdelims (){n}}^\frac{1}{n}|K_n(g_{K,L})|^\frac{1}{n}={{2n}\atopwithdelims (){n}}^\frac{1}{n}\frac{|K|^\frac{1}{n}|L|^\frac{1}{n}}{M(K,L)^\frac{1}{n}}\ge {{2n}\atopwithdelims (){n}}^\frac{1}{n}\frac{|K|^\frac{1}{n}|L|^\frac{1}{n}}{\min \{|K|^\frac{1}{n},|L|^\frac{1}{n}\}}\\= & {} {{2n}\atopwithdelims (){n}}^\frac{1}{n}\max \{|K|^\frac{1}{n},|L|^\frac{1}{n}\}\ge \frac{1}{2}{{2n}\atopwithdelims (){n}}^\frac{1}{n}(|K|^\frac{1}{n}+|L|^\frac{1}{n}). \end{aligned}$$

Assume that there is equality for some $\left( \frac{3}{4}\right) ^n\le \theta _0<1$. Then, by the equality cases in Theorem 1.2, we have that

$$\begin{aligned} g_{K,L}(x)=M(K,L)\left( 1-\Vert x\Vert _{K+L}\right) ^n\quad \forall x\in K+L. \end{aligned}$$

Otherwise, we have that for every $0\le t\le \frac{1}{4}$

$$\begin{aligned} t{{2n}\atopwithdelims (){n}}^\frac{1}{n}K_n(g_{K,L})\subsetneq L_{1-t}(g_{K,L}). \end{aligned}$$

or, equivalently, taking $t=1-\theta ^\frac{1}{n}$ we have that for every $\left( \frac{3}{4}\right) ^n\le \theta <1$

$$\begin{aligned} {{2n}\atopwithdelims (){n}}^\frac{1}{n}K_n(g_{K,L})\subsetneq \frac{L_{\theta ^\frac{1}{n}}(g_{K,L})}{1-\theta ^\frac{1}{n}}=\frac{K+_\theta L}{1-\theta ^\frac{1}{n}}. \end{aligned}$$

and then, in particular,

$$\begin{aligned} \left| \frac{K+_{\theta _0} L}{1-\theta _0^\frac{1}{n}}\right| ^\frac{1}{n}>{{2n}\atopwithdelims (){n}}^\frac{1}{n}|K_n(g_{K,L})|^\frac{1}{n}\ge \frac{1}{2}{{2n}\atopwithdelims (){n}}^\frac{1}{n}(|K|^\frac{1}{n}+|L|^\frac{1}{n}), \end{aligned}$$

which contradicts the equality at $\theta _0$.

Therefore, if there is equality for some $\left( \frac{3}{4}\right) ^n\le \theta _0\le 1$, we have that for every $0\le \theta <1$

$$\begin{aligned} K+_\theta L=(1-\theta ^\frac{1}{n})(K+L) \end{aligned}$$

and then, as mentioned in Sect. 2.3, $K=-L$ is a simplex.

For any $0\le \theta \le \left( \frac{3}{4}\right) ^n$ we have that $0\le \theta ^\frac{1}{n}\le \frac{3}{4}$ and

$$\begin{aligned} \theta ^\frac{1}{n}=\left( \frac{4}{3}\theta ^\frac{1}{n}\right) \frac{3}{4}+\left( 1-\frac{4}{3}\theta ^\frac{1}{n}\right) 0. \end{aligned}$$

Since $g_{K,L}^\frac{1}{n}$ is concave on $K+L$, we have that

$$\begin{aligned} K+_\theta L= & {} L_{\theta ^\frac{1}{n}}(g_{K,L})\supseteq \frac{4}{3}\theta ^\frac{1}{n}L_{\frac{3}{4}}(g_{K,L})+\left( 1-\frac{4}{3}\theta ^\frac{1}{n}\right) L_0(g_{K,L})\\\supseteq & {} \frac{1}{3}\theta ^\frac{1}{n}{{2n}\atopwithdelims (){n}}^\frac{1}{n}K_n(g_{K,L})+\left( 1-\frac{4}{3}\theta ^\frac{1}{n}\right) (K+L), \end{aligned}$$

where the last inclusion relation is a consequence of Theorem 1.2. Taking volumes and using Brunn–Minkowski inequality we obtain that

$$\begin{aligned} |K+_\theta L|^\frac{1}{n}\ge & {} \frac{1}{3}\theta ^\frac{1}{n}{{2n}\atopwithdelims (){n}}^\frac{1}{n}|K_n(g_{K,L})|^\frac{1}{n}+\left( 1-\frac{4}{3}\theta ^\frac{1}{n}\right) |K+L|^\frac{1}{n}\\\ge & {} \frac{1}{3}\theta ^\frac{1}{n}{{2n}\atopwithdelims (){n}}^\frac{1}{n}\frac{|K|^\frac{1}{n}|L|^\frac{1}{n}}{M(K,L)^\frac{1}{n}}+\left( 1-\frac{4}{3}\theta ^\frac{1}{n}\right) \left( |K|^\frac{1}{n}+|L|^\frac{1}{n}\right) \\\ge & {} \frac{1}{3}\theta ^\frac{1}{n}{{2n}\atopwithdelims (){n}}^\frac{1}{n}\max \{|K|^\frac{1}{n},|L|^\frac{1}{n}\}+\left( 1-\frac{4}{3}\theta ^\frac{1}{n}\right) \left( |K|^\frac{1}{n}+|L|^\frac{1}{n}\right) \\\ge & {} \frac{1}{6}\theta ^\frac{1}{n}{{2n}\atopwithdelims (){n}}^\frac{1}{n}\left( |K|^\frac{1}{n}+|L|^\frac{1}{n}\right) +\left( 1-\frac{4}{3}\theta ^\frac{1}{n}\right) \left( |K|^\frac{1}{n}+|L|^\frac{1}{n}\right) \\= & {} \left( 1-\left( \frac{4}{3}-\frac{1}{6}{{2n}\atopwithdelims (){n}}^\frac{1}{n}\right) \theta ^\frac{1}{n}\right) \left( |K|^\frac{1}{n}+|L|^\frac{1}{n}\right) . \end{aligned}$$

$$\square $$

Finally, let us point out that, as a consequence of the estimates obtained in the proof of Theorem 1.1, we can obtain another proof of inequality (2.3):

### Corollary 4.1

Let $K,L\subseteq {\mathbb {R}}^n$ be a pair of convex bodies such that $M(K,L)=|K\cap (-L)|$. Then

$$\begin{aligned} |C_1(K,L)|^\frac{1}{n}\ge \frac{1}{n}{{2n}\atopwithdelims (){n}}^\frac{1}{n}\frac{|K|^\frac{1}{n}|L|^\frac{1}{n}}{M(K,L)^\frac{1}{n}}, \end{aligned}$$

with equality if and only if $K=-L$ is a simplex.

### Proof

We have seen in the previous proof that for every $\left( \frac{3}{4}\right) ^n\le \theta <1$

$$\begin{aligned} \left| \frac{K+_\theta L}{1-\theta ^\frac{1}{n}}\right| ^\frac{1}{n}\ge {{2n}\atopwithdelims (){n}}^\frac{1}{n}\frac{|K|^\frac{1}{n}|L|^\frac{1}{n}}{M(K,L)^\frac{1}{n}}. \end{aligned}$$

Taking limit as $\theta \rightarrow 1^-$, since $\frac{K+_\theta L}{1-\theta ^\frac{1}{n}}$ is an increasing family of convex bodies in $\theta$ such that

$$\begin{aligned} \lim _{\theta \rightarrow 1^-}\frac{K+_\theta L}{1-\theta ^\frac{1}{n}}=\lim _{\theta \rightarrow 1^-}\frac{1-\theta }{1-\theta ^\frac{1}{n}}\frac{K+_\theta L}{1-\theta }=nC_1(K,L), \end{aligned}$$

we obtain the result. Moreover, if $K=-L$, the inequality above becomes Zhang’s inequality (2.2), where there is equality if and only if $K=-L$ is a simplex. On the other hand, if there is equality, by the equality case in Theorem 1.2, then necessarily $g_{K,L}=M(K,L)(1-\Vert x\Vert _{K+L})^n$. Therefore, for every $\theta \in [0,1]$ we have that

$$\begin{aligned} K+_\theta L=(1-\theta ^\frac{1}{n})(K+L) \end{aligned}$$

and then $K=-L$ is a simplex. $\square$
